# Comparison of saline infusion test and captopril challenge test in the diagnosis of Chinese with primary aldosteronism in different age groups

**DOI:** 10.3389/fendo.2024.1343704

**Published:** 2024-03-22

**Authors:** Kaiwen Sun, Minghui Gong, Yang Yu, Minghui Yang, Ying Zhang, Yinong Jiang, Wei Song

**Affiliations:** Department of Cardiology, First Affiliated Hospital of Dalian Medical University, Dalian, Liaoning, China

**Keywords:** primary aldosteronism, saline infusion test, captopril challenge test, age, Chinese

## Abstract

**Background:**

To explore the diagnostic accuracy and the optimal cutoff value between the saline infusion test (SIT) and captopril challenge test (CCT) [including the value and suppression of plasma aldosterone concentration (PAC)] for primary aldosteronism (PA) diagnosing.

**Methods:**

A total of 318 patients with hypertension were consecutively enrolled, including 126 patients with PA and 192 patients with essential hypertension (EH), in this observational study. The characteristics of patients and laboratory examinations were collected and compared. The comparison between SIT and CCT was carried by drawing the receiver operator characteristic curve (ROC) and calculating the area under the curve (AUC) to explore the diagnostic accuracy and the optimal cutoff value.

**Results:**

The average age was 51.59 ± 10.43 in the PA group and 45.72 ± 12.44 in the EH group (*p*<0.05). The optimal cutoff value was 10.7 ng/dL for post-CCT PAC, 6.8 ng/dL for post-SIT PAC, and 26.9% for suppression of post-CCT PAC. The diagnostic value of post-CCT PAC was the highest with 0.831 for the AUC and 0.552 for the Youden index. The optimal cutoff value for patients who were <50 years old was 11.5 ng/dL for post-CCT PAC and 8.4 ng/dL for post-SIT PAC. The suppression of post-CCT PAC turned to 18.2% for those of age 50 or older.

**Conclusion:**

Compared with SIT, CCT had a higher diagnostic value when post-CCT PAC was used as the diagnostic criterion in Chinese people, while the selection of diagnostic thresholds depended on patient age.

## Introduction

Primary aldosteronism (PA), a common cause of secondary hypertension, occurs in 5%–10% of patients with hypertension, with higher ratios in those with resistant hypertension (1–3). Its pathogenesis is related to the increased secretion of autonomic aldosterone in one or both adrenal cortical globular zones, which causes water and sodium retention, leading to increased circulatory loading and blood pressure (4, 5). Recent research has shown that a higher incidence of cardiovascular events and more severe target organ damage are observed in PA compared with essential hypertension (EH) (3, 6–9). Therefore, early diagnosis and treatment of PA are of great significance.

According to the 2016 Endocrine Society guidelines, patients considered to have a possible PA diagnosis based on preliminary screening need to undergo confirmatory testing (10). There are four confirmatory tests for PA with diverse strengths and limitations: the fludrocortisone suppression test, the oral saline load test, the saline infusion test (SIT), and captopril challenge test (CCT). SIT and CCT are currently in wide clinical use due to their convenience and affordability (10, 11). However, some debates still remain among previous studies (12). There has not been a clear result comparing the accuracy of these confirmatory tests in diagnosing PA. There were several cutoff values in different guidelines (13–15). The interpretation of the results can be affected by factors such as the discrepant daily sodium intake in various countries (16). In addition, cutoff values are not fixed among various ethnic groups and ages (17–19). Leung et al. (20) pointed out that there were significant differences in the interpretation and verification of the results of the confirmatory tests, and there had been almost no effective reference standard to test at present, which made it difficult to distinguish.

The aim of our study is to compare the diagnostic efficiency between SIT and CCT and calculate the optimal cutoff value in different age groups among Chinese people to improve the diagnostic accuracy for PA.

## Materials and methods

### Study population

There were 2,546 patients diagnosed with hypertension who were admitted to the First Affiliated Hospital of Dalian Medical University in January 2019 and June 2021 and 346 patients aged 18–80 suspected with PA in the First Affiliated Hospital of Dalian Medical University were consecutive enrolled and performed SIT and CCT after drug eluting. Angiotensin-converting enzyme inhibitor (ACEI), angiotensin receptor blocker (ARB), dihydropyridine calcium channel blocker (CCB), and β receptor blockers were stopped for at least 2 weeks, diuretics for at least 4 weeks, and aldosterone receptor antagonists for at least 6 weeks to eliminate the interference of drugs to the results. The antihypertensive drugs were chosen as α receptor blockers or non-dihydropyridine calcium channel blockers for blood pressure controlling. Sodium intake was not restricted. A total of 318 eligible patients were enrolled according to their symptoms, signs, and specific results of examination referring to the latest guideline (10).

Exclusion criteria were as follows: (1) other types of secondary hypertension [such as renal parenchymal hypertension, severe renal artery stenosis, Cushing’s syndrome, pheochromocytoma, and severe obstructive sleep apnea (OSA)], white coat hypertension, pseudo hypertension, etc.; (2) severe liver or renal damage such as aspartate aminotransferase (AST) or alanine aminotransferase (ALT) more than three times of the upper limit of normal and estimated glomerular filtration rate (eGFR) <60 ml/min * 1.73 m^2^; (3) heart failure with left ventricular ejection fraction (LVEF) <50%; (4) history of malignancy; (5) women taking contraceptives recently or pregnancy; and (6) those with mental and intellectual disorders and patients who refused to join the study.

### Clinical characteristic

The characteristics of patients were collected including age, gender, body mass index (BMI), duration of hypertension, history of smoking, and drinking.

### Biochemistry measurement

The laboratory examinations were collected from fasting serum for at least 8 h and urine for the first in the morning and throughout the day of patients. The fasting serum index included ALT, AST, creatinine (Cr), uric acid (UA), serum sodium and potassium, glycosylated hemoglobin (HbA1c), fasting plasma glucose (FPG), total cholesterol (TC), triglyceride (TG), high-density lipoprotein cholesterol (HDL-C), low-density lipoprotein cholesterol (LDL-C), and type B natriuretic peptide (BNP). The urine index included 24 h urine sodium and potassium throughout the day. eGFR was calculated by CKD-EPI formula (21, 22). All laboratory examinations mentioned before were measured by automated biochemical instrument.

### Screening and confirmatory test

Direct renin concentration (DRC) and plasma aldosterone concentration (PAC) in vertical and horizontal positions were measured by chemiluminescence (DiaSorin S.P.A, Saluggia, Italy). The diagnosis of PA relied on a combination with symptoms, signs, a positive ARR (≥3.7 ng/dL per mU/L), and one or more positive confirmatory tests following the guideline (10).

1. Screening test: patients were required to maintain a non-vertical (sitting or standing) position for at least 2 h after getting up in the morning. The horizontal DRC and PAC was collected after sitting for 5–15 min. The ratio of plasma aldosterone to renin concentration (ARR) was calculated by PAC/DRC, and the test would be considered positive when ARR ≥ 3.7 ng/dL per mU/L (10).
2. SIT: SIT was carried out at 8:00 a.m. with the intravenous infusion of 2 L of 0.9% sodium chloride solution for a 4-h test. DRC, PAC, and ARR before and after infusion were measured. The test is considered positive when post-SIT PAC was more than 10 ng/dL, whereas it was negative when post-SIT PAC was <5 ng/dL (10).
3. CCT: patients remained in a sitting position, and 50 mg captopril was administered orally. DRC, PAC, and ARR were measured before CCT and 1 and 2 h after taking the captopril. The standard included the PAC measurement value and PAC suppression at 2 h post-administration. The test was considered positive when post-CCT PAC was >11 ng/dl or the suppression of post-CCT PAC was <30% (10).

### Statistical analysis

SPSS 24.0 software was used for statistical analysis. When comparing the differences between two groups, the data that conformed to the normal distribution and met the homogeneity of variance were analyzed by t-test; the data that conformed to the normal distribution but did not meet the homogeneity of variance were analyzed by the corrected t-test. The results above were described by mean ± standard deviation. The data that did not conform to the normal distribution were analyzed by Mann–Whitney U-test and were described by median and quartile. The counting data were analyzed by χ^2^ test and were described by percentage. The comparison between SIT and CCT was carried by drawing the receiver operator characteristic curve (ROC) and calculating the area under the curve (AUC) to find the optimal cutoff value. Any *p <*0.05 was considered statistically significant.

## Results

### Clinical characteristic of patients

**Figure 1:**
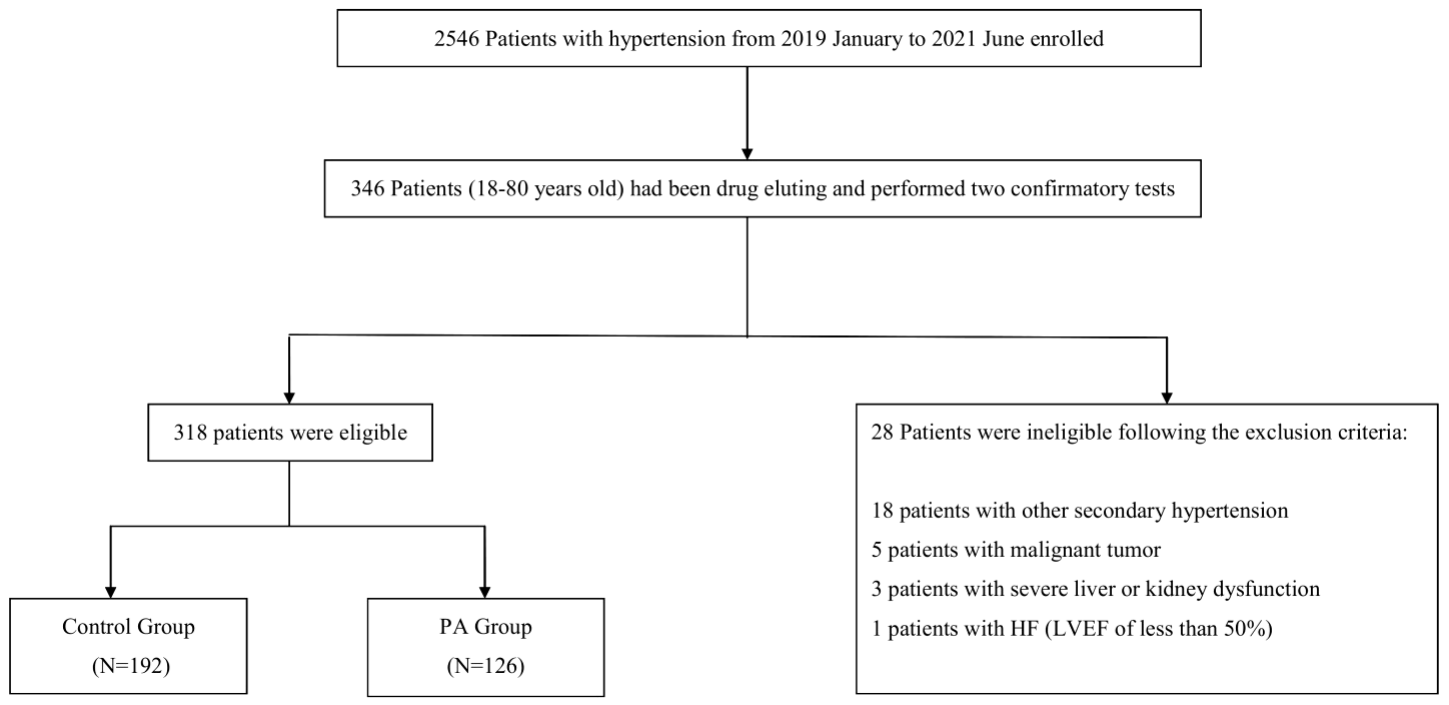
Flow chart of the study.

A total of 318 patients were consecutively enrolled, including 126 patients with PA and 192 patients with EH (**Figure 1**). The average age was 51.45 ± 10.48 years in the PA group and 45.84 ± 12.37 years in the EH group (*p* < 0.05). The patients in the PA group presented with longer hypertension duration and lower BMI than the EH group (*p* < 0.05). There was no difference between the two groups with respect to the proportion of smokers, alcohol history, or sex distribution (*p* > 0.05). Meanwhile, the concentration of serum potassium without supplementation before ARR screening was lower, and the concentration of serum sodium and 24-h urine potassium was higher in the PA group than in the EH group (*p* < 0.05). The PA group also had lower DRC and higher PAC compared with the EH group (*p* < 0.05). BNP and HDL-C was higher, and ALT, AST, TG and UA was lower in the PA group, although these laboratory test results were within normal ranges (*p* < 0.05) (**Table 1**).

**Table 1:** Clinical characteristic of patients.

	PA group (n=126)	EH group (n=192)	*p*
Age (years)	51.45 ± 10.48	45.84 ± 12.37	<0.001*
Male (N,%)	55(43.7%)	95(49.5%)	0.309
BMI (kg/m^2^)	26.28 ± 3.67	27.46 ± 4.06	0.009*
Duration of hypertension (years)	7.00(1.00, 10.25)	2.50(0.33, 7.00)	<0.001*
History of smoking (N, %)	25(19.8%)	43(22.4%)	0.587
History of drinking (N, %)	23(18.3%)	47(24.5%)	0.190
Laboratory examinations			
ALT (U/L)	17.00(13.00, 25.25)	24.00(15.25, 33.00)	<0.001*
AST (U/L)	16.00(14.00, 20.25)	18.00(16.00, 23.00)	0.001*
Cr (mmol/L)	63.56 ± 16.04	66.21 ± 16.45	0.157
eGFR (mL/min/1.73m^2^)	102.07 ± 13.70	104.60 ± 14.26	0.117
BNP (ng/L)	34.08(18.34,56.94)	20.65(10.97, 34.08)	<0.001*
Na^+^ (mmol/L)	142.46 ± 2.58	141.33 ± 2.01	<0.001*
K^+^ (mmol/L)	3.51 ± 0.46	3.71 ± 0.33	<0.001*
FPG (mmol/L)	4.73(4.42, 5.19)	4.71(4.33, 5.25)	0.784
HbA1c (%)	5.60(5.40, 5.80)	5.60(5.40, 5.90)	0.415
TC (mmol/L)	4.61 ± 0.86	4.76 ± 0.99	0.171
TG (mmol/L)	1.25(0.94,1.71)	1.50(1.02,2.19)	0.002*
HDL-C (mmol/L)	1.16 ± 0.28	1.08 ± 0.26	0.013*
LDL-C (mmol/L)	2.51 ± 0.63	2.66 ± 0.72	0.050
UA (μmol/L)	327.56 ± 82.31	368.80 ± 92.83	<0.001*
Horizontal DRC (mU/L)	1.21(0.50, 3.24)	4.32(1.83, 12.69)	<0.001*
Horizontal PAC (ng/dL)	17.0 ± 9.2	11.9 ± 5.4	<0.001*
Vertical DRC (mU/L)	4.03(1.69, 7.79)	12.50(4.92, 29.24)	<0.001*
Vertical PAC (ng/dL)	24.8 ± 12.9	19.5 ± 9.2	<0.001*
24 h urine sodium (mmol/24 h)	143.37 ± 68.65	141.29 ± 83.31	0.816
24 h urine potassium (mmol/24 h)	54.77 ± 23.58	42.05 ± 18.40	<0.001*

PA, primary aldosteronism; BMI=body mass index; SBP, systolic blood pressure; DBP, diastolic blood pressure; ALT, alanine aminotransferase; AST, aspartate aminotransferase; Cr, creatinine; eGFR, estimated glomerular filtration rate; BNP, type B natriuretic peptide; FPG, fasting plasma glucose; HbA1c, glycated hemoglobin A1c; TC, total cholesterol; TG, triglyceride; HDL-C, high-density lipoprotein cholesterol; LDL-C, low-density lipoprotein cholesterol; UA, uric acid; DRC, direct renin concentration; PAC, plasma aldosterone concentration.

*p <0.05.

### Diagnostic efficacy of SIT and CCT by guideline

According to the diagnostic criteria in the guideline (10), 34 of 126 patients (26.7%) in the PA group had positive SIT results, and eight patients (6.3%) had negative results. The rest (66.7%) had indeterminate results (5–10 ng/dL). In the EH group, 44 of 192 patients (22.9%) were SIT negative and three (1.6%) were SIT positive. The remaining 75.5% were indeterminate. For CCT, the post-CCT PAC was positive in 102 of 126 patients (81.0%) in the PA group, whereas 137 of 192 patients (71.4%) in the EH group had negative post-CCT PAC. When using the suppression of post-CCT PAC as the criterion, 109 of 126 PA group patients (86.5%) were CCT positive, and 97 of 192 EH group patients (50.5%) were CCT negative (**Table 2**).

**Table 2:** The specific number of patients in each confirmatory test.

Confirmatory tests	PA(n=126)	EH(n=192)	Sensitivity/specificity	Youden Index
Post-CCT PAC (+)	102	55	81.0%/71.4%	0.524
Post-CCT PAC (−)	24	137		
Post-SIT PAC (+)	34	3	27.0%/22.9%	–
Post-SIT PAC (−)	8	44		
Post-SIT PAC in gray gap	84	145		
Suppression of post-CCT PAC(+)	109	95	86.5%/50.5%	0.370
Suppression of post-CCT PAC(−)	17	97		

PA, primary aldosteronism; EH, essential hypertension; CCT, captopril challenge test; PAC, plasma aldosterone concentration; SIT, saline infusion test.

### Diagnostic value of SIT and CCT

**Figure 2:**
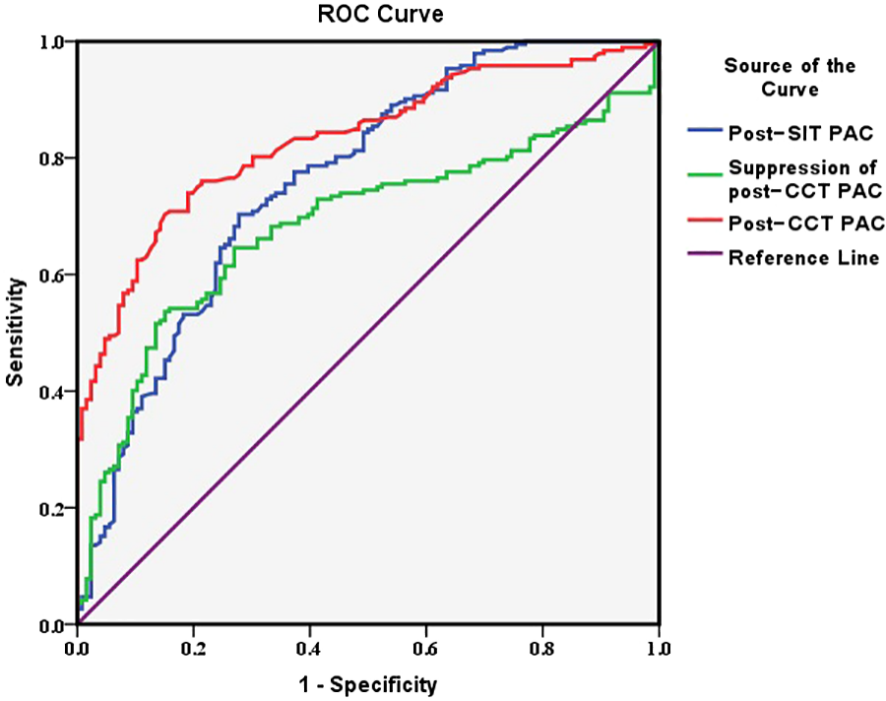
The ROC curve of each confirmatory test for PA among all ages.

The optimal cutoff values were 10.7 ng/dL for post-CCT PAC, 6.8 ng/dL for post-SIT PAC, and 26.9% for post-CCT PAC suppression. The diagnostic value of post-CCT PAC was the highest, with an AUC of 0.831 [95% CI: (0.787, 0.875)] and Youden index of 0.552. For SIT, the AUC was 0.762 [95% CI: (0.708, 0.816)] and the Youden index was 0.425. There was a lowest diagnostic value of the suppression of post-CCT PAC, with an AUC of 0.684 [95% CI: (0.625, 0.743)] and Youden index of 0.385 (**Table 3**) (**Figure 2**).

**Table 3:** Diagnostic value of each confirmatory test.

Confirmatory Tests	Cutoff value	AUC (95%CI)	Sensitivity	Specificity	Youden Index
Post-CCT PAC (ng/dL)	10.7	0.831 (0.787,0.875)	84.9%	70.3%	0.552
Post-SIT PAC (ng/dL)	6.8	0.762 (0.708,0.816)	72.2%	70.3%	0.425
Suppression of post-CCT PAC	26.9%	0.684 (0.625,0.743)	53.6%	84.9%	0.385

AUC, area under the curve; CCT, captopril challenge test; PAC, plasma aldosterone concentration; SIT, saline infusion test.

### Diagnostic value between SIT and CCT in different age groups

**Figure 3:**
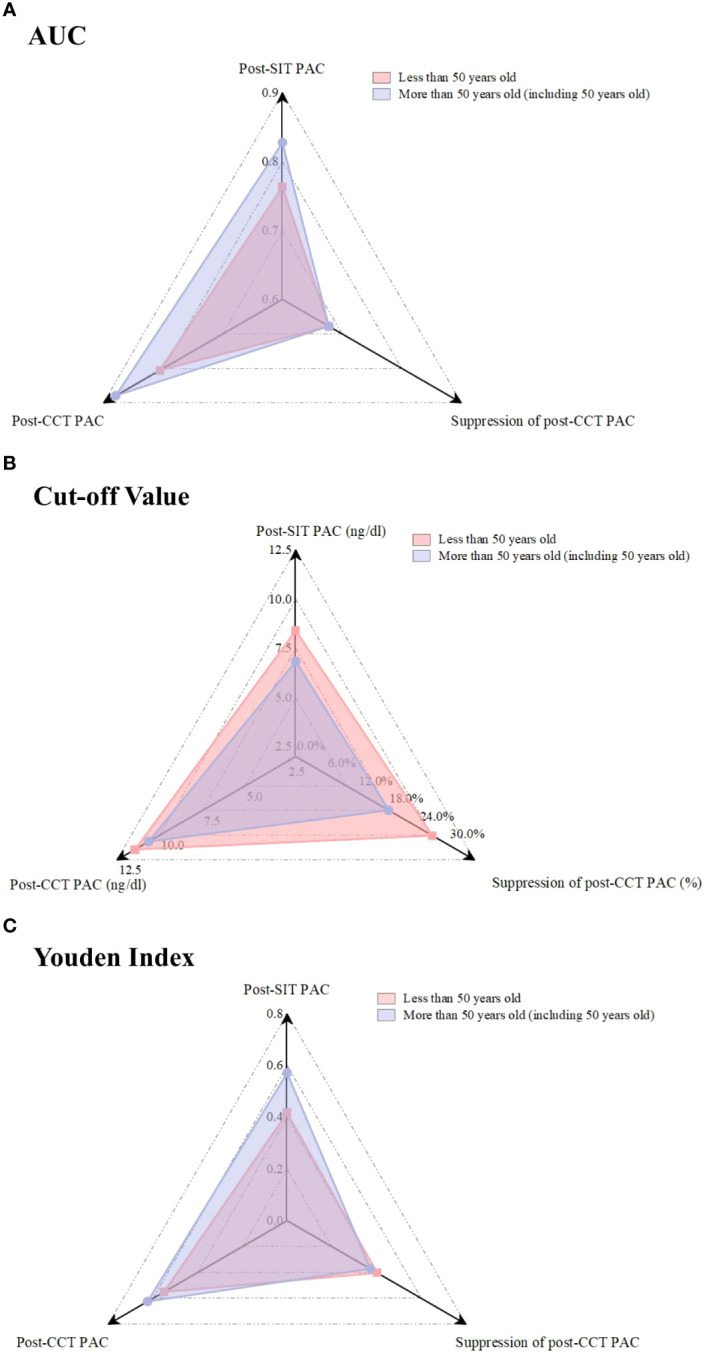
Comparison of the AUC, Cut-off value, and Youden Index for each confirmatory test in different age.

The patients were further divided into two groups according to age. The post-CCT PAC showed the greatest diagnostic value, with a higher AUC and Youden index regardless of age group (**Figures 3A–C**). As for the optimal cutoff values of each confirmatory test, it was increased to 11.5 ng/dL for post-CCT PAC and 8.4 ng/dL for post-SIT PAC among those who were <50 years old. The suppression of post-CCT PAC was 26.8%, which was similar to that mentioned above. For patients who were age 50 or older, the post-CCT PAC suppression was 18.2%, while the values of post-CCT PAC and post-SIT PAC did not change.

## Discussion

It is necessary to identify, diagnose, and treat with PA in a timely manner to control blood pressure and reduce the risk of related complications. The consensus and guidelines for the diagnosis and treatment of PA have been continually updated for decades. Although the diagnostic process came to be gradually simplified, there was a wide overlap in ARR values between normokalemic patients with PA and those with EH, which need a caution if skipping the confirmatory test according to the research (23). The confirmatory test is also the key evidence for a clear diagnosis of PA and reduces unnecessary risks, such as invasive diagnosis and/or surgical treatment for patients when it is accurate and reliable (10, 11). The present relevant guideline recommends four confirmatory tests (10). However, the sensitivity and specificity of each confirmatory test vary. It lacks sufficient evidence to recommend any single confirmatory test as the best one. Nowadays, confirmatory test selection is generally based on cost, patient compliance, local hospital conditions, etc. Considering multiple study factors, SIT and CCT are widely used and compared for PA diagnosis to select the appropriate method and identify their diagnostic values in Chinese people of different ages.

SIT and CCT had different mechanisms of action. SIT inhibited renin and aldosterone secretion via volume overload, while CCT suppressed aldosterone secretion by decreasing the angiotensinase activity and increasing the renin level. Thus, the diagnostic criteria of the two tests were inconsistent (24, 25). SIT had a high prevalence of gray zone in the PA and EH groups according to the criteria in the guideline, which were unable to clarify PA temporarily and required further confirmatory tests (10). Thus, the necessity for a definite diagnostic threshold in SIT was recognized, which was found to be controversial in previous studies (18, 26–28). In this study, the optimal cutoff value was 6.8 ng/dL for the post-SIT PAC. The post-SIT PAC was collected in various (sitting or horizontal) positions in different studies, and various detection methods, such as immunoassays or liquid chromatography coupled with tandem mass spectrometry, were used, which might explain the varied results (29, 30). The latest research recommended doubling the upper limit of salt intake in Chinese people (16, 31). A high salt intake could decrease aldosterone secretion through negative feedback regulation, leading to lower reactivity for SIT and aldosterone detection (32). The cutoff value in this study was below the one that the guidelines proposed, which might be attributed to higher salt intake according to the 24-h urine sodium test. For CCT, the oral dose of captopril, the difference in position, individual drug metabolisms when collecting the serum, and even the reference diagnostic criteria were inconsistent, leading to non-unified results (33, 34). In this study, the cutoff value was 10.7 ng/dL for post-CCT PAC and 26.9% for the suppression of post-CCT PAC, which was close to the guideline recommendation (10).

The comparison between the tests showed that all of them were feasible. The diagnostic efficacy of post-CCT PAC was relatively reliable, with a larger AUC and a higher Youden index, which was consistent with previous studies (18, 27, 29). The CCT was relatively simple and quick to operate. Additionally, it did not require infusion facilities and was more suitable for older patients with relatively high blood pressure (5, 35). Therefore, it might be more practicable when diagnosing with PA.

There was lack of a sufficient understanding of PA among senior citizens. One of the reasons was that there was a high incidence of cardiovascular disease with multiple drug treatment that could not withdraw these drugs among them, which influenced the screening test results and made performing the confirmatory test challenging (36). However, the resistant hypertension proportion and cardiovascular disease complications increased with age (37). Renin concentrations also decreased with age. Therefore, it is important to screen for PA among older populations. This study included a relatively wide range of patient ages, which might have impacted the results. Kuo et al. (38) showed that the average age at PA diagnosis was approximately 50 by reviewing previous studies. Therefore, the group was divided at age 50 to explore diagnostic accuracy and the optimal cutoff value. The optimal post-SIT PAC and post-CCT PAC cutoff values for patients who were less than 50 years old increased. The renin–angiotensin–aldosterone system (RAAS) activity decreased among older people, leading to the hyposecretion of aldosterone (39). Therefore, the cutoff value was lower than that in younger ones. The diagnostic value was highest, had the largest AUC, and had a high Youden index when using the post-CCT PAC in each group, further proving the superiority of CCT.

This study had some limitations. First, this was an observational study, and some biases might exist. Second, the sample size was relatively small, and the patients were from a single center. Larger patient samples were required. Third, the study lacked details on patient management and follow-up. We collected the data on follow-up in PA group of which after the subtype differentiation and underwent medication treatment or surgery as shown in the **Supplemental Table S1**. The data of patients who underwent surgery and followed up were shown in the **Supplementary Tables S2** and **S3**. We note that 11 patients reached biochemical complete success, and three patients reached clinical complete success in accordance with the Primary Aldosteronism Surgical Outcome (PASO) criteria (40). However, the rate of follow-up was relatively low. The incidence of PA in this study was nearly 5%, according to our statistics. In fact, there were about one-fifth of patients with elevated ARR but negative confirmatory test developed overt PA over time (41). We found that nearly one-third patients with a positive ARR but negative confirmatory tests in EH group. PA is a biochemical continuous process, and these patients may be in the early stages of the disease or in a subclinical state, requiring closer follow-up in the future to get a correct diagnosis at an earlier stage of the disease. In addition, the research showed that aldosterone levels were independently related to the degree of OSA and that PA and OSA interacted (42). To avoid the impact of severe OSA on the results, these patients with PA were excluded. Thus, the substantial proportion was even higher. Finally, some biochemical indices, such as HDL-C and UA, differed between the two groups, which previous studies also observed, and the mechanism requires further exploration (43, 44).

## Conclusion

Compared with SIT, CCT had a higher diagnostic value when post-CCT PAC was used as the diagnostic criterion in Chinese people, while the selection of diagnostic thresholds depended on patient age.

## Data availability statement

The raw data supporting the conclusions of this article will be made available by the authors, without undue reservation.

## Ethics statement

The studies involving humans were approved by the Ethics Committee of the First Affiliated Hospital of Dalian Medical University. The studies were conducted in accordance with the local legislation and institutional requirements. The participants provided their written informed consent to participate in this study. Written informed consent was obtained from the individual(s) for the publication of any potentially identifiable images or data included in this article.

## Author contributions

KS: Software, Methodology, Investigation, Formal analysis, Data curation, Conceptualization, Writing – review & editing, Writing – original draft. MG: Writing – review & editing, Software, Methodology, Formal analysis, Data curation. YY: Writing – review & editing, Visualization, Software, Methodology. MY: Writing – review & editing, Project administration. YZ: Writing – review & editing, Resources. YJ: Writing – review & editing, Validation. WS: Writing – review & editing, Supervision.
