# Supplementary material for: Comparison of saline infusion test and captopril challenge test in the diagnosis of Chinese with primary aldosteronism in different age groups
Source: Front Endocrinol (Lausanne). 2024 Mar 22;15:1343704. doi: 10.3389/fendo.2024.1343704 (PMC10995348; doi:10.3389/fendo.2024.1343704)
Supplement: Supplementary file 1 [file Table_1.doc]

Supplemental Table 1 The data of follow-up in patients with PA

| Laboratory tests | Before Treatment | After Treatment | *P* |
| --- | --- | --- | --- |
| Na+ (mmol/L) | 142.44±2.97 (n=68) | 140.76±2.22 (n=68) | <0.001* |
| K+ (mmol/L) | 3.52±0.47 (n=96) | 4.08±0.37 (n=96) | <0.001* |
| Vertical DRC (uIU/ml) | 5.19(1.75,11.50) (n=23) | 8.32(3.46,15.27) (n=23) | 0.156 |
| Vertical PAC (ng/dl) | 23.5(17.7, 35.5) (n=23) | 10.0 (7.3,23.1) (n=23) | 0.001* |

DRC= direct renin concentration, PAC= plasma aldosterone concentration **P*<0.05

Supplemental Table 2 The data of follow-up in patients with PA before and after surgery

| Laboratory tests | Before Surgery (N=12) | After Surgery (N=12) | *P* |
| --- | --- | --- | --- |
| K+ (mmol/L) | 3.47±0.49 | 4.28±0.30 | <0.001* |
| Vertical DRC (uIU/ml) | 3.46(1.85,6.60) | 9.85(4.46,28.40) | 0.017* |
| Vertical PAC (ng/dl) | 25.1(17.8, 35.4) | 8.3(4.9,15.0) | <0.001* |

DRC= direct renin concentration, PAC= plasma aldosterone concentration **P*<0.05

**Supplemental Table 3 Comparison** of clinical and biochemical outcomes in patients with PA

|  | Number of the patients (N=12) |
| --- | --- |
| **Clinical outcomes** |  |
| Complete success | 3 (25.0%) |
| Partial success | 9 (75.0%) |
| Absent success | 0 |
| **Biochemical outcomes** |  |
| Complete success | 11 (91.7%) |
| Partial success | 1 (8.3%) |
| Absent success | 0 |
